# Supplementary material for: Prostate-Specific Membrane Antigen (PSMA)-Positive Extracellular Vesicles in Urine—A Potential Liquid Biopsy Strategy for Prostate Cancer Diagnosis?
Source: Cancers (Basel). 2022 Jun 17;14(12):2987. doi: 10.3390/cancers14122987 (PMC9221222; doi:10.3390/cancers14122987)
Supplement: Supplementary file 1 [file cancers-14-02987-s001.zip › cancers-1774050-supplementary.pdf]

Supporting information for:

Prostate-specific membrane antigen (PSMA) -positive extracellular vesicles in urine - a potential liquid biopsy strategy for prostate cancer diagnosis?

Susann Allelein[1]*, Keshia Aerchlimann[1], Gundula Rösch[2], Roxana Khajehamiri[2], Andreas Kölsch[1], Christian Freese[2], Dirk Kuhlmeier[1]

[1] Affiliation: Fraunhofer Institute for Cell Therapy and Immunology (IZI) Leipzig, Germany
[2] Affiliation: Fraunhofer Institute for Microengineering and Microsystems (IMM) Mainz, Germany
* Correspondence: susann.allelein@izi.fraunhofer.de;

Keywords: extracellular vesicles, prostate specific membrane antigen, microarray, immunomagnetic isolation, automated, prostate cancer

Table S1. Cohort characteristics of PCa patients and benign male controls. The risk group corresponds to the Gleason Score (GS) determined after prostate tissue biopsy.

RISK	#	AGE	PSA [ng/mL]	CREATININE [mmol/L]	GS	PROSTATE V [mL]
**benign**	1	56	14.11	23.99		126
**benign**	2	53	20.20	14.04		63
**benign**	3	66	3.89	2.55		70
**benign**	4	67	2.99	0.38		100
**benign**	5	78	0.95	0.62		24
**benign**	6	74	3.50	2.45		87
**benign**	7	74	6.00	7.03		50
**benign**	8	70	10.00	5.08		90
**benign**	9	66	2.25	20.17		40
**benign**	10	68	7.40	11.75		120
**benign**	11	58	11.70	18.31		70
**benign**	12	72	0.93	2.25		130
**benign**	13	56	8.00	32.66		70
**benign**	14	66	5.05	30.77		51
**benign**	15	78	5.50	7.78		75
**benign**	16	68	17.70	4.70	6	128
**low**	1	66	9.79	5.34		110
**low**	2	54	1.10	6.33		60
**low**	3	77	2.60	5.75		50
**low**	4	56	5.00	15.94	6	30
**low**	5	62	7.00	6.48	6	30
**intermediate**	1	55	12.20	14.21	7a	25
**intermediate**	2	68	5.86	13.97	7a	70
**intermediate**	3	67	12.60	7.26	7a	120

**intermediate**	4	63	12.90	8.99	6	60
**intermediate**	5	77	7.67	13.66	7a	30
**intermediate**	6	56	4.80	18.46	7	40
**intermediate**	7	75	12.70	20.40	7	15
**intermediate**	8	73	4.20	10.77	7	18
**intermediate**	9	78	6.10	10.78	7	10
**intermediate**	10	74	5.00	8.92	7	35
**intermediate**	11	63	5.12	15.59	7	40
**intermediate**	13	62	5.29	7.97	7	66
**intermediate**	14	76	8.44	8.86	7	45
**intermediate**	15	63	14.50	6.36	7	55
**high**	1	58	6.60	20.48	7	25
**high**	2	67	7.30	3.9	8	94
**high**	3	71	6.80	15.58	9	60
**high**	4	62	6.71	3.95	9	30
**high**	5	69	34.50	3.01	9	240
**high**	6	71	5.40	1.21	9	20
**high**	7	67	28.00	1.92	8	77

Table S2. Cohort characteristics of healthy female and male controls.

#	SEX	#	AGE	CREATININE [µmol/L]
**1**	female	1	35	24850
**2**	female	2	27	24730
**3**	female	3	30	1603
**4**	female	4	34	1963
**5**	female	5	32	1176
**6**	male	6	22	2724
**7**	male	7	36	2552
**8**	male	8	29	3950
**9**	male	9	29	21782
**10**	male	10	29	16070

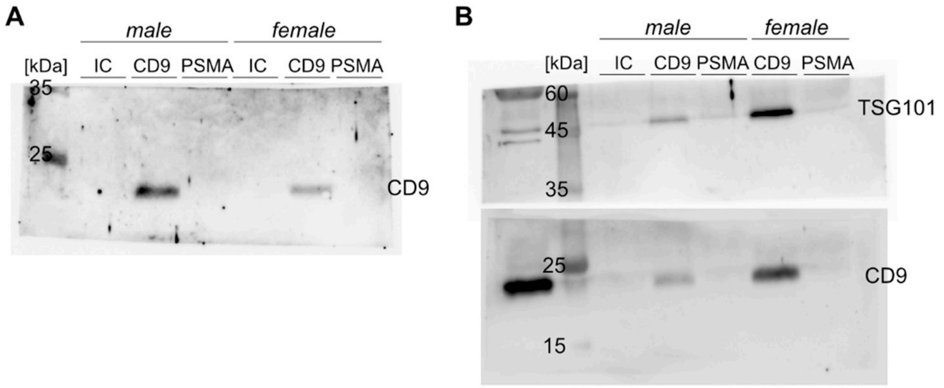

Figure S1. Specific uEV isolation using immunomagnetic beads. Western blot analysis for CD9 and/or TSG101 of CD9- or PSMA-positive uEVs and the isotype control (IC) from female and male intermediate PCa risk urine from patient intermediate 15 (**A**) and 3 (**B**).

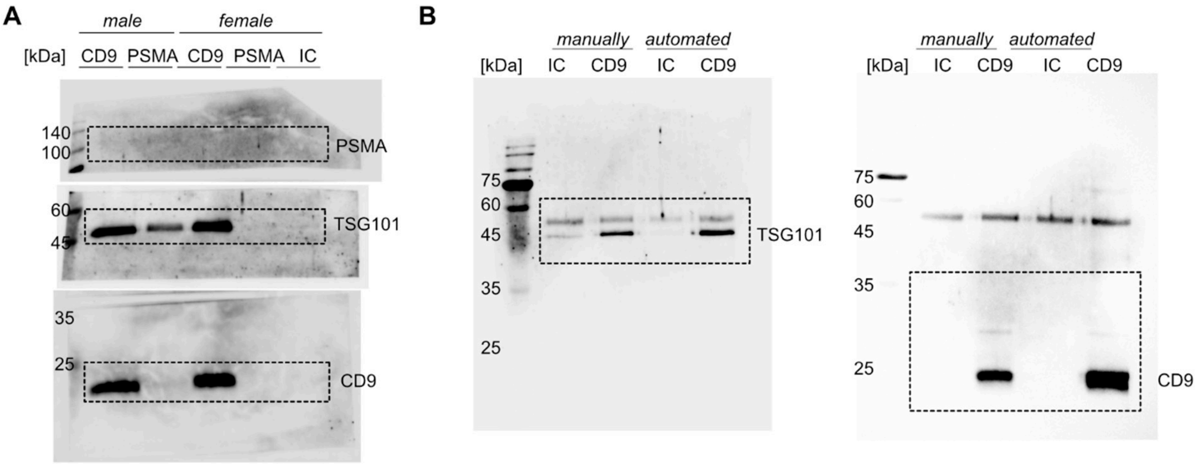

Figure S2. Western blot images for specific uEV isolation using immunomagnetic beads with cropped areas indicated by dashed lines used in figure 2. Antibody incubation on membrane cuts according to the molecular weight of the investigated protein.

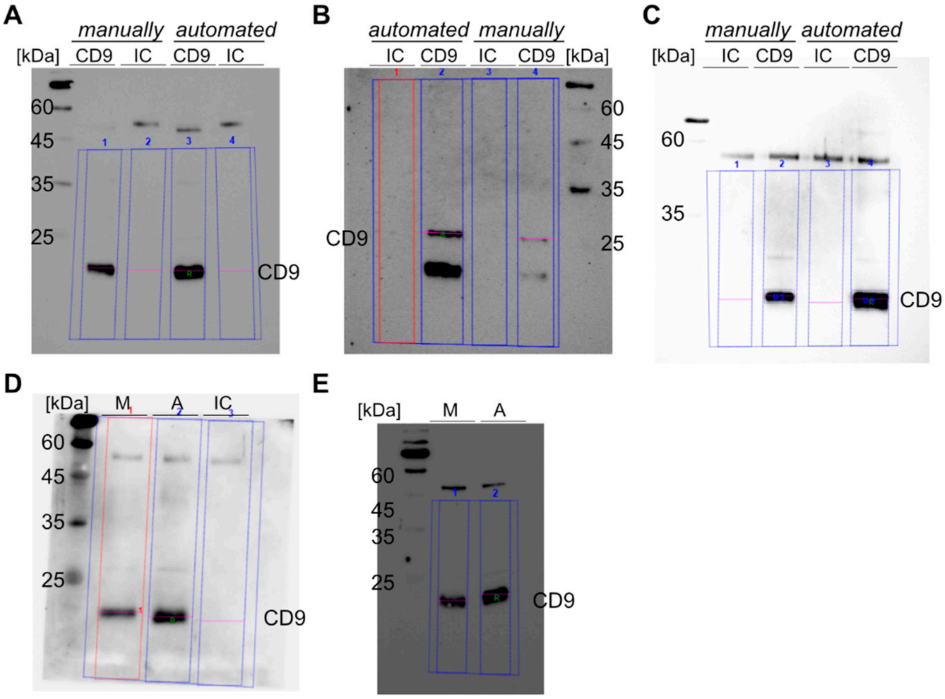

Figure S3. Western blot analysis of CD9 from specific uEV immunomagnetic isolation by manually (M) and automated (A) performed procedure targeting the isotype control (IC) or CD9 (**A** -**C**) or a mix of CD63 and CD81 (**D**, **E**) from 5 mL of cell-free urine. Relative band intensities of CD9 analyzed in Image Lab software (Bio-Rad Laboratories) and summarized in Fig. 2.

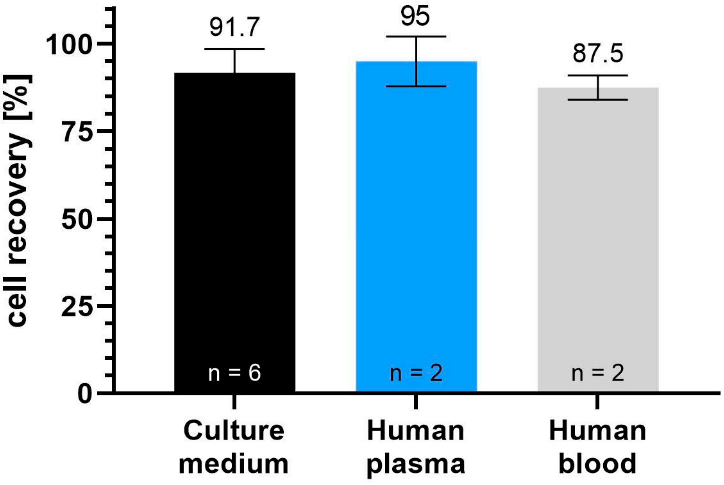

Figure S4. Characterization of the IsoMAG-ONE10.0 isolation system. For testing the functionality and the optimal isolation parameters, such as number of washing steps, buffers used, mixing times and volumes, enrichment of cells from cell culture medium, human plasma and whole blood was performed. For this purpose, 20 fluorescently stained cells were added to the enrichment medium and the cells were automatically enriched with EpCAM-coated Dynabeads. After enrichment, the cell number of the enriched cells was determined and the percentages calculated in relation to the 20 previously added cells. This protocol did not only show best enrichment properties but also lowest loss of beads during the process which is of importance for the isolation of EVs as demonstrated in Fig. 3.

Table S3. Assay parameters for IsoMAG-ONE10.0 (duration: 1:34 h)

No.	Step	Description
**1**	Reset all axes	Required
**2**	Take pipette	Take 10ml pipette
**3**	Move to well 1	5ml + 100 µl *Beads* in 6ml tube
**4**	Prepare pipette	Pump 0,5ml air
**5**	Dive in pipette	Dive in fluid
**6**	Mixing	30min; 20ml/min
**7**	Get *Beads*	Magnet to pipette and release fluid; 60s
**8**	Move to well 2	4ml in 6ml tube
**9**	Prepare pipette	Pump 0,5ml air
**10**	Dive in pipette	Dive in fluid
**11**	Peel off	10x; 20ml/min; 1,1ml
**12**	Washing	20x ; 20ml/min
**13**	Get *Beads*	Magnet to pipette and release fluid; 60s
**14**	Move to well 3	4ml in 6ml tube
**15**	Prepare pipette	Pump 0,5ml air
**16**	Dive in pipette	Dive in fluid
**17**	Peel off	10x; 20ml/min; 1,1ml
**18**	Washing	20x ; 20ml/min
**19**	Get *Beads*	Magnet to pipette and release fluid; 60s
**20**	Move to well 4	1ml in 2ml tube
**21**	Prepare pipette	Pump 0,5ml air

**22**	Dive in pipette	Dive in fluid
**23**	Washing	25x ; 20ml/min
**24**	Release Fluid	Release fluid from pipette without magnet
**25**	Drop pipette	Drop 10ml pipette
**26**	Take pipette	Take 1,25ml pipette
**27**	Move to well 4	1ml in 2ml tube
**28**	Prepare pipette	Pump 0,5ml air
**29**	Dive in pipette	Dive in fluid
**30**	Mixing	5x; 5ml/min
**31**	Get *Beads*	Magnet to pipette and release fluid; 60s
**32**	Move to well 5	0,2ml in 1,9 ml tube
**33**	Prepare pipette	Pump 0,5ml air
**34**	Dive in pipette	Dive in fluid
**35**	Washing	30x ; 2 ml/min
**36**	Release Fluid	Release fluid from pipette without magnet
**37**	Drop pipette	Drop 1,25ml pipette
